# Intraosseous Atypical Verrucopapillary Epithelial Proliferation with Verrucous Carcinoma Features Evolving from a Keratinizing Odontogenic Cyst: Report of an Uncommon Variant and Review of the Literature

**DOI:** 10.1007/s12105-026-01890-7

**Published:** 2026-02-02

**Authors:** Kristie L. Wise, Kristin K. McNamara, John R. Kalmar, Prokopios P. Argyris

**Affiliations:** 1 Division of Oral and Maxillofacial Pathology, The Ohio State University College of Dentistry, Postle Hall, Room 2191, 305 W. 12th Ave, Columbus, OH 43210 USA; 2 CTA Oral Pathology, 300 Marconi Blvd., Suite 308, Columbus, OH 43215 USA; 3 Department of Pathology, The University of Chicago Medicine, 5841 S. Maryland Avenue, MC 6040, Chicago, IL 60637 USA

**Keywords:** Intraosseous verrucous carcinoma, Developmental odontogenic cyst, Atypical verrucopapillary epithelial proliferation, Keratinizing odontogenic cyst, Intraosseous squamous cell carcinoma

## Abstract

**Background:**

Developmental odontogenic cysts (DOCs) of the jawbones with overt luminal keratinization, i.e., odontogenic keratocyst (OKC) and orthokeratinized odontogenic cyst (OOC), are relatively uncommon in head and neck/oral pathology practice. Notably, atypical verrucopapillary epithelial proliferations (AVPEP) arising from the epithelium of DOCs have rarely been reported and range from verrucous epithelial hyperplasia with or without cytologic dysplasia to verrucous carcinoma (VC). Published examples of intraosseous VC developing ex-DOC are exceptionally limited, previously making assessment of their biologic behavior and design of optimal therapy uncertain.

**Materials and methods:**

A 37-year-old man with a history of non-Hodgkin lymphoma of the right neck presented with right posterior maxillary sensitivity of at least 6-years’ duration. Radiographically, a partially-corticated, unilocular radiolucency was identified enveloping the roots of the first and second right maxillary molars causing buccal and palatal cortical expansion. The maxillary lesion was excised along with extraction of involved teeth.

**Results:**

Histopathologic examination showed a cystic lesion lined by markedly acanthotic stratified squamous epithelium exhibiting a verrucopapillary architecture with broad, bulbous, rete ridges, prominent luminal parakeratin production and parakeratin cleft formation. Occasional intraepithelial keratin whorls were also present. Cytologic aberrations including basilar crowding, increased nuclear-to-cytoplasmic ratio, hyperchromatism and precocious keratinization were observed. The cyst wall comprised chronically inflamed fibrous tissue. Definite mural invasion was not identified in multiple sections. By immuhistochemistry, p16 staining was patchy, while p53 and Ki67 decorated basal and, focally, suprabasal cells. No recurrence was reported after 14 months of follow-up.

**Conclusion:**

Here, we report an uncommon case of intraosseous AVPEP with VC features developing from the epithelial lining of a keratinizing DOC. Despite their alarming histopathologic appearance, a review of the literature reveals that such lesions tend to have a favorable prognosis following complete surgical excision.

## Introduction

Developmental odontogenic cysts (DOCs) are relatively common in oral and maxillofacial pathology practice with most of them lacking evidence of luminal keratinization [1, 2]. Luminal keratin production in DOCs is most commonly identified in odontogenic keratocyst (OKC; previously keratocystic odontogenic tumor) [2–5] and orthokeratinized odontogenic cyst (OOC) [6–8], but can be infrequently encountered in cystic lesions demonstrating combined histopathologic characteristics of otherwise distinct subtypes of DOCs, e.g., OKC with OOC [9] and OOC or OKC with calcifying odontogenic cyst (Gorlin cyst) [10–12]. Overall, diagnosis of OKC and OOC is rather straightforward for oral/head and neck pathologists familiar with their distinctive features [13]. Diagnostic challenges may arise when keratinizing DOCs are secondarily inflamed, leading to partial or complete effacement of their characteristic histomorphology [1, 4, 13].

There are reports of keratinizing DOCs presenting with exuberant and, occasionally, verrucous hyperkeratosis that may exhibit marked cytologic and architectural atypia [14, 15] and, in certain instances, even fulfill the diagnostic criteria of verrucous carcinoma (VC) [16–20]. Owing to their rarity, the biologic behavior of such atypical verrucopapillary epithelial proliferations (AVPEP), i.e., verrucous hyperplasia and VC, arising in the setting of DOCs remains poorly-documented and optimal treatment has remained uncertain. Here, we report the clinicopathologic characteristics of an additional example of intraosseous AVPEP evolving from the epithelial lining of a keratinizing DOC displaying features compatible with VC. In addition, a comprehensive review of the pertinent literature is performed with emphasis on prognosis and optimal therapy.

## Materials and Methods

### Literature Search

Publicly available electronic databases, including PubMed, Medline and Google Scholar, were searched for previously reported cases of AVPEPs arising from DOCs using the following combination of keywords: “verrucous carcinoma”, “papillary proliferation”, “verrucous/verrucoid proliferation”, “verrucous hyperplasia”, or “architectural atypia” and “odontogenic cyst”, “developmental odontogenic cyst”, or “odontogenic keratocyst”. Specific diagnostic criteria were applied to identify potential cases of interest. AVPEPs ex-odontogenic cyst were defined as epithelial cystic lesions demonstrating a marked verrucopapillary architecture with or without the concomitant presence of cytologic atypia or dysplasia. By definition, AVPEPs in the setting of DOCs encompass verrucous hyperplasia, VC, as seen in the current example, and anecdotally papillary SCC without *bona fide* invasive conventional SCC. Acceptable examples of VC ex-odontogenic cyst exhibited an overt verrucopapillary morphology with prominent hyperkeratosis, keratin clefting, marked acanthosis, and thickened/broad rete ridges “pushing” into the fibrous wall, in conjunction with minimal or mild cytologic atypia. Finally, epithelial dysplasia within the cystic lining of a DOC comprises atypical architectural and/or cytologic features, including a verrucous or papillary architecture, expanded proliferative compartment, i.e., basilar crowding, increased N:C ratio, nuclear pleomorphism, nuclear hyperchromasia, visible macronucleoli, and brisk mitotic activity.

Inclusion criteria for the current review comprised case reports published in the English-written literature with adequate documentation of the clinical and histopathologic features of the lesion. By definition, examples of primary intraosseous squamous cell carcinoma (SCC) with histologic features of conventional SCC or carcinoma cuniculatum arising ex-DOC were excluded. Eleven previous reports fulfilling the above criteria were identified [14, 16–25]. Patient age and sex, lesion size, location and radiographic findings, as well as treatment and follow-up information were retrieved and tabulated.

### Case Presentation

#### Clinical and Radiographic Features

**Fig. 1:**
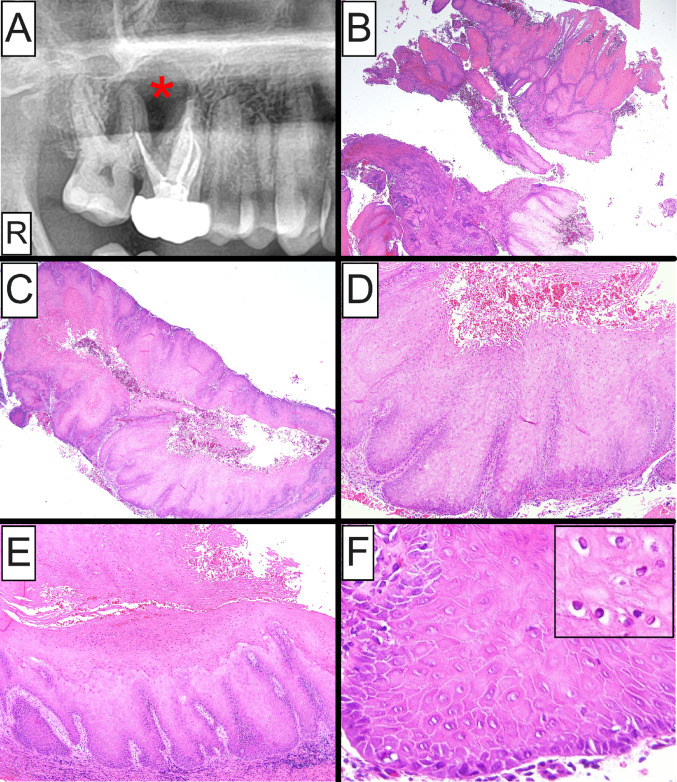
Clinical and histopathologic characteristics of intraosseous VC-like atypical verrucopapillary epithelial proliferation arising in the setting of a keratinizing odontogenic cyst. **A** Panoramic radiograph showing a partially corticated, unilocular radiolucency of the right posterior maxilla (asterisk); **B, C** Low-power photomicrographs showing a fragmented cystic lesion featuring prominent acanthosis with hyperparakeratosis and marked verrucous architecture of the epithelial lining; **D, E** Medium-power photomicrographs exhibiting broad and bulbous rete ridges of the epithelial lining akin to VC with prominent luminal parakeratin production and parakeratin clefting; **F** High-power photomicrograph showing features of maturational disorganization, including increased nuclear-to-cytoplasmic ratio, basilar crowding, nuclear hyperchromasia, and precocious keratinization limited to the basilar one-third of the epithelial thickness; koilocyte-like changes of superficial keratinocytes were noted (**F inset**)

A 37-year-old man presented with a chief complaint of sensitivity in the right posterior maxilla of at least 6-years’ duration. Root canal treatment of the right maxillary first molar (tooth #3) failed to alleviate the symptoms. His medical history was significant for non-Hodgkin lymphoma (Grade III; Stage 1) of the right neck treated with R-CHOP (rituximab, cyclophosphamide, doxorubicin, vincristine, prednisone) chemotherapy and field radiation 14 years prior. Radiographically, a partially corticated, unilocular radiolucency was identified in the right posterior maxilla involving the roots of teeth #2 and #3 (Fig. 1A), causing palatal and buccal cortical expansion. An excisional biopsy was performed along with extraction of the involved teeth and a clinical impression of odontogenic keratocyst.

#### Histologic and Immunohistochemical Findings

At gross examination the submitted specimen comprised multiple, tannish-brown, soft tissue fragments measuring 4.1 × 1.8 × 0.7 mm in aggregate. Histopathologic examination revealed a fragmented cystic lesion featuring prominent verrucous architecture of the epithelial lining with keratin plugging and occasional parakeratin whorl formation (Fig. 1B, C). Marked acanthosis with elongated, bulbous and confluent rete ridges was observed, together with marked luminal parakeratin production and parakeratin clefting (Fig. 1D, E). Cytologic epithelial aberrations included basilar crowding, nuclear pleomorphism and hyperchromasia, increased nuclear-to-cytoplasmic ratio and precocious keratinization (Fig. 1F). These dysplastic alterations were generally limited to the basilar one-third of the overall epithelial thickness. No definitive evidence of stromal invasion was identified on multiple sections. In areas, superficial keratinocytes with pyknotic dark-staining nuclei and optically clear cytoplasm, akin to koilocytes, were noted (**F inset**). The cyst wall was composed of dense fibrous connective tissue supporting a moderate diffuse influx of predominantly chronic inflammatory cells.

**Fig. 2:**
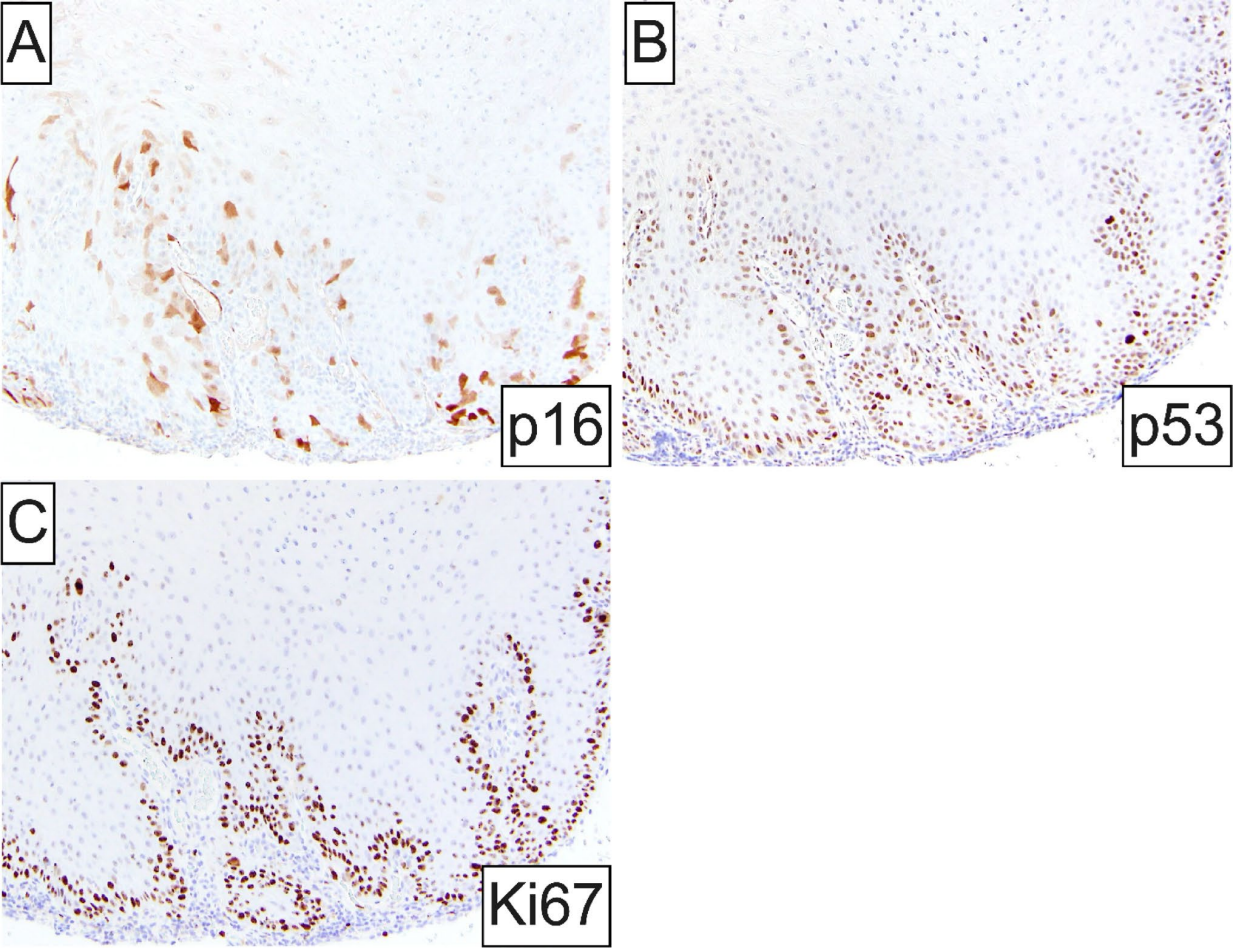
Immunohistochemical findings: **A** Patchy, discontinuous immunopositivity for the high-risk HPV surrogate marker p16; **B, C** nuclear staining for p53 and Ki67 proliferation index is limited to the basal and suprabasilar compartment of the atypical verrucous cystic epithelium

By immunohistochemistry, lesional cells showed patchy only immunoreactivity for p16 (Fig. 2A), indicating the absence of transcriptionally active high-risk HPV infection. Proliferative index Ki-67 expression and p53 staining were generally limited to the basal and suprabasilar compartments of the atypical verrucous cystic epithelium (Fig. 2B, C), consistent with wild-type p53.

#### Follow-Up

A diagnosis of keratinizing odontogenic cyst with mild cytologic dysplasia and architectural disorganization was rendered with a comment underscoring the uncommon histopathologic features of this lesion and histomorphologic similarities to VC, together with a recommendation for close clinical follow-up. No additional treatment was rendered and no evidence of recurrence has been detected in 14 months of follow-up with subsequent panoramic imaging.

## Discussion

AVPEPs arising in the setting of keratinizing DOCs are rare lesions with only N = 11 previous examples reported in the English literature (Table 1) [14, 16–25]. When the current case is also considered, 10 (83.3%) affected men and 2 (16.7%) women (M:F ratio = 5:1) with a mean age of 50.5 years (age range = 13–76 years). A predilection for the maxilla was noted (8 of 12, 66.7%) with only 4 cases involving the mandible. The anterior and posterior portions of the jaws were equally affected. Accompanying clinical findings included symptomatic or painless, expansile, intraosseous lesions with occasional overlying mucosal involvement, cutaneous or intraoral sinus tracts (fistulas) [16, 17, 21] and an average duration of 39.4 months (range 6–96 months). Radiographically, most lesions presented as well-defined, unilocular or multilocular radiolucencies, with or without cortical thinning and perforation, and an average size of 3.4 cm (range 2.0–8.3 cm). A single lesion was described as radiopaque and extended into the maxillary sinus [24]. Infrequent complications include tooth mobility [17], trismus [21] and lower lip/chin paresthesia [23].

**Table 1:** Clinicoepidemiologic characteristics of previously reported intraosseous atypical verrucopapillary epithelial proliferations arising from odontogenic cystic lesions

Author (Year)	Age (years)/sex	Location/size (cm)	Clinico-radiographic presentation	Diagnosis	Treatment	Follow-up (months)/outcome
Enriquez et al. [21]	56/M	R posterior mandible 2.0 × 2.0	Painless, well-defined RL causing tonsillar pillar swelling, cutaneous fistulas and trismus of at least 3-months duration**	VC arising from odontogenic cyst	En bloc resection	48; NED
Anand et al. [22]	46/M	R anterior maxilla 2.0 × 2.0	Painful, well-defined, expansile RL of 6-months duration	VC arising from odontogenic keratocyst with synchronous SCC	Curettage with subsequent partial maxillectomy and nasal septectomy	24; NED
Pomatto et al. [16]	NA/F	R posterior maxilla NA	Painless RL causing mucosal fistula and swelling of 36-months duration	VC arising from odontogenic cyst	Surgical excision	8; NED
Aldred et al. [25]	13/F	R anterior maxilla NA	Asymptomatic, expansile, unilocular RL of less than 24-months duration**	Verrucous proliferation arising from odontogenic cyst	Enucleation	NA; NED
Ueeck et al. [23]	46/M	L posterior mandible NA	Multilocular RL causing lower lip and chin paresthesia	Atypical verrucous proliferation arising from keratinizing odontogenic cyst	Enucleation and segmental mandibulectomy upon recurrence	7; recurrence 27; NED
Mohtasham et al. [18]	58/M	R anterior maxilla 3.5 × 2.5	Painless, well-defined RL with cortical perforation and mucosal involvement of 48-months duration	VC arising from odontogenic keratocyst	Enucleation	20; NED
Imaue et al. [24]	52/M	L posterior maxilla 4.6 × 2.6*	Painless, well-defined RO with maxillary sinus involvement, cortical perforation and mucosal involvement, developing from unilocular RL of 10 years prior**	VC arising from odontogenic cyst	Partial maxillectomy	NA; NED
Peng et al. [17]	74/M	L anterior mandible 3.5 × 1.9	Mildly painful, expansile, well-defined RL causing tooth mobility and purulent discharge of 6-months duration	VC arising from dentigerous cyst	Surgical excision	5; NED
Argyris et al. [14]	32/M	L posterior mandible 8.3 × 4.0	Asymptomatic, well-defined, multilocular RL causing cortical thinning	Verrucous hyperplasia with epithelial dysplasia arising from keratinizing odontogenic cyst	Surgical excision	66; NED
Kamarthi et al. [20]	65/M	L anterior maxilla 2.8 × 2.0	Painful, well-defined RL causing cortical thinning, perforation and multifocal mucosal involvement of 12-months duration	VC arising from orthokeratinized odontogenic cyst	Surgical excision	6; NED
Kang and Leem [19]	76/M	R anterior maxilla 2.0 × 2.0 and 1.0 × 1.0*	Painless, well-defined RL progressing to ill-defined RL causing cortical perforation, multifocal mucosal and skeletal muscle involvement of 96-months duration	VC arising from odontogenic cyst	Partial maxillectomy and neck dissection	24; NED
Present case (2025)	37/M	R posterior maxilla 2.0	Painful, well-defined expansile RL of 72-months duration	VC arising from keratinizing odontogenic cyst	Surgical excision	14; NED

VC, verrucous carcinoma; RL, radiolucency; RO, radiopacity; NED, no evidence of disease; NA, not available

*These measurements represent the soft tissue portion of the intraosseous lesion and do not reflect the actual size of the lesion. Therefore, they have been excluded from the calculation of the mean size

**The exact duration of the lesion was not specified. While recorded here, these times were not included in the final calculation of mean duration

The histopathologic spectrum of AVPEPs evolving from the epithelial lining of DOCs is broad and may include a markedly acanthotic, hyperplastic epithelium with hypergranulosis, hyperortho- or parakeratosis, together with a verrucous or papillary architecture and inconspicuous to pronounced cytologic atypia [14, 23, 25]. More frequently, however, AVPEPs ex-DOC demonstrate features akin to VC of surface epithelial origin [16–22, 24], as also seen in the present case. Such VC-like cystic lesions of the jawbones show exuberant luminal keratin production with keratin plug formation, as well as elongated, bulbous and broad rete ridges “pushing” into the fibrous cyst wall. Cytologic atypia, when present, is usually limited to the basal/suprabasilar layers. Koilocyte-like features, i.e., keratinocytes with pyknotic nuclei and clear perinuclear halo, although present, does not correlate with HPV infection, as confirmed by negative HPV PCR testing and patchy p16 immunoreactivity [14, 15]. Notably, tautochronous occurrence of AVPEP and intraosseous SCC evolving from a DOC has also been documented [22].

**Fig. 3:**
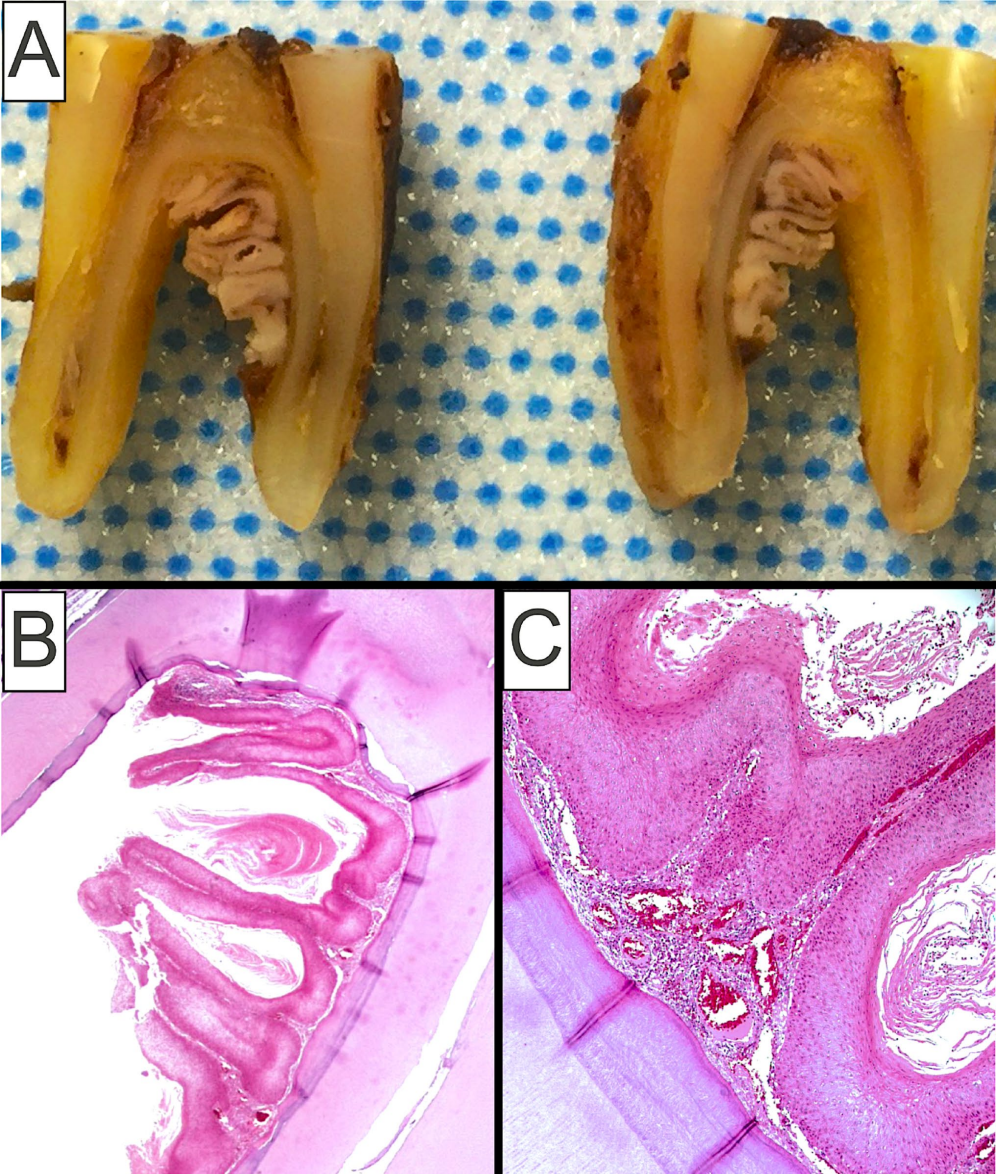
**A** Gross images of proliferative verrucous leukoplakia lesion infiltrating the periodontal ligament space (PDL) and extending into the underlying alveolar bone at the root bifurcation of this mandibular molar tooth; **B, C** Histologic characteristics of proliferative verrucous leukoplakia involving the PDL space comprising an atypical epithelial proliferation with verrucoid architecture, prominent parakeratin production and clefting, together with cytologic aberrations (Images courtesy of Dr. Ioannis Koutlas, DDS, MS, University of Minnesota)

Two main differential diagnoses necessitate attention when an intraosseous cystic AVPEP ex-DOC is considered. Odontogenic cysts with a verrucoid pattern should be distinguished from proliferative verrucous leukoplakic lesions that have infiltrated the periodontal ligament space extending into the alveolar bone proper and causing cyst-like saucerization of the bone (Fig. 3A–C) [13]. Such lesions can be clinically and even microscopically misinterpreted as AVPEPs arising from an intraosseous odontogenic cyst (Fig. 3B, C). A useful diagnostic feature, however, is the presence of clinically detectable marginal linear gingival leukoplakia usually in the form of a “*ring around the collar*” of involved teeth [26, 27]. Additionally, well-differentiated cystic SCC, including carcinoma cuniculatum, can masquerade as a jawbone DOC with luminal keratinization, particularly in incisional or small biopsy specimens [28, 29]. Notably, thorough microscopic examination of the entirely submitted specimen of AVPEP, including VC, arising in the lining of a DOC is mandatory to exclude the tautochronous presence of conventional SCC.

**Fig. 4:**
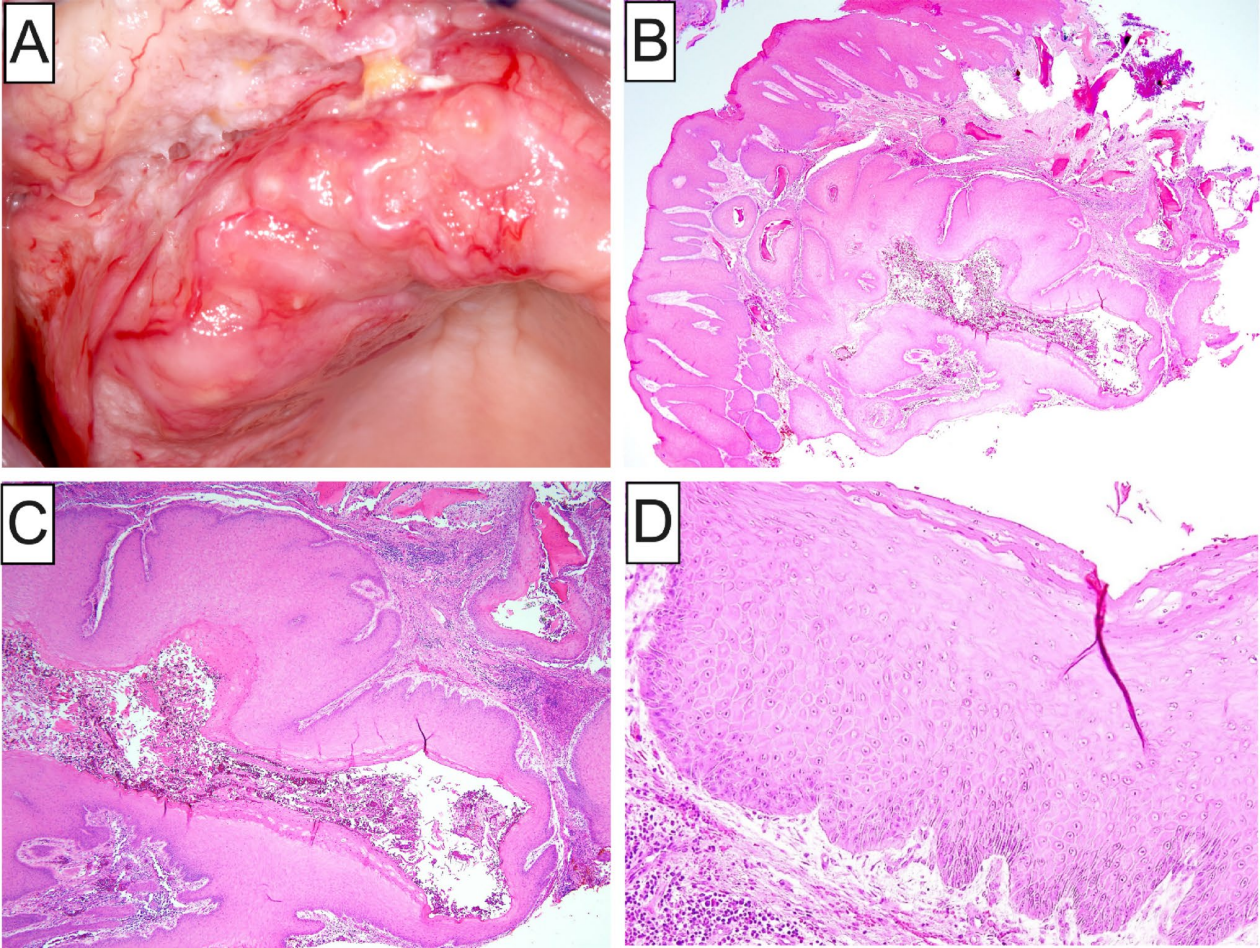
**A** Clinical presentation of carcinoma cuniculatum involving the maxilla, as well as maxillary alveolar and labial mucosa causing buccal and palatal cortical expansion and superficial telangiectasia; **B, C** Low-power photomicrographs showing an endophytic growth pattern comprising a burrowing network of well-differentiated squamous epithelium forming interconnecting keratin-filled crypts with exuberant amounts of exfoliated keratin and neutrophilic microabscesses; **D** Cytologic atypia in carcinoma cuniculatum is at most mild

Carcinoma cuniculatum deserves special mention due to its, overall, bland cytomorphology and overlapping clinical and histologic characteristics with AVPEPs developing in the setting of DOCs. Carcinoma cuniculatum demonstrates a strong predilection for the jawbones (Fig. 4A) and a predominantly endophytic growth pattern comprising a burrowing labyrinthine network of well-differentiated squamous epithelium forming interconnecting keratin-filled crypts invading the underlying bone (Fig. 4B, C) [28, 30–33]. As a result, microsequestra are frequently present. The epithelial crypts display exuberant amounts of exfoliated keratin in association with neutrophilic microabscesses (Fig. 4C). Similar to this case of atypical VC-like proliferation evolving from a DOC, epithelial dysplasia is at most mild in carcinoma cuniculatum of the jawbones (Fig. 4D) and a verrucopapillary architecture may be, at least focally, identified (Fig. 4C). In contrast to keratinizing odontogenic cysts with verrucous features, carcinoma cuniculatum is characterized by multiple, infiltrative, cyst-like crypts, as well as architectural and/or cytologic aberrations of the overlying mucosal epithelium (Fig. 4B). However, these two diagnostically helpful findings may not be identified owing to non-representative tissue sampling.

Treatment of the AVPEPs in the setting of DOCs has varied greatly (Table 1). Eight (66.7%) patients, including the current case, were treated conservatively, i.e., surgical excision, enucleation, or curettage, while 4 (33.3%) received radical surgical resection and one of them additional neck dissection [19]. The single case of VC with tautochronous SCC arising from the epithelial lining of an OKC was treated with curettage followed by partial maxillectomy and nasal septectomy [22]. Loco-regional recurrence was reported in only 1 of 12 (8.3%) patients within 7 months after enucleation [23]. The remaining 11 patients (91.7%) with AVPEP evolving from a DOC showed no evidence of disease after a mean follow-up period of 24.2 months (range = 5–66 months), notwithstanding conservative therapeutic regimens in most cases. Given the favorable prognosis with conservative therapy, extensive resection should be reserved for recurrent or locally aggressive lesions, or when a synchronous SCC component is identified.

## Conclusion

Herein, we report the clinicopathologic and immunophenotypic characteristics of a rare example of intraosseous AVPEP fulfilling diagnostic criteria for VC and evolving from the keratinizing epithelium of a DOC. Taking into consideration that only a few similar cases have been reported, such VC-like epithelial cystic lesions of the jawbones show a strong predilection for the maxilla of middle-aged men. Despite their alarming histologic and, occasionally, clinico-radiographic appearance, they have generally been associated with a favorable prognosis even after conservative surgical management, although periodic clinical and radiographic follow-up would appear warranted.

## Data Availability

Available clinical, histopathologic and immunohistochemical data pertaining to the current case are presented in the manuscript. Original files are available upon reasonable request from the corresponding author.
